# Drug Information Sources for Patients with Chronic Conditions in the Qassim Region, Saudi Arabia

**DOI:** 10.3390/pharmacy11020057

**Published:** 2023-03-16

**Authors:** Saeed Alfadly, Mohammed Anaam, Mohammed Alshammari, Saud Alsahali, Ejaz Ahmed, Abdulkareem Bin Mubarak, Abdullah Aldahouk, Muhanna Aljameeli

**Affiliations:** 1 Department of Pharmacy Practice, Unaizah College of Pharmacy, Qassim University, P.O. Box 5888, Unaizah 51911, Qassim, Saudi Arabia; 2 Department of Pharmacy, College of Medicine and Health Sciences, Hadramout University, Al Mukalla P.O. Box 8892, Yemen

**Keywords:** drug information, chronic illness, Qassim Region, medication adherence

## Abstract

Appropriate drug information is vital for the correct use of drugs in pharmaceutical practice. Providing patients with educational advice on prescribed medication and on proper medication administration has become an essential part of the pharmaceutical care process. The objectives of this study were to identify patients’ knowledge of prescribed medications, their desire for more information, and the sources of medication information in a population from Qassim, Saudi Arabia, using a cross-sectional descriptive study. Our target population consisted of adult patients with chronic illnesses receiving drugs at outpatient pharmacies. Nineteen pharmacies were selected based on convenience. After collecting their prescriptions, patients were asked to take part in the study by interviewers as they were leaving the pharmacies. The questionnaire used was pretested on 18 patients and then modified accordingly. questions investigated participants’ knowledge of drug information, their wish for more information, and their sources of drug information, other than clinicians. Descriptive analysis was used to describe patients’ physical details. The effect of sex, education, diagnosis, number of drugs, and age on knowledge of the purpose of drugs and the need for additional information were tested using Chi-square test. A total of 270 patients were interviewed, of whom 29.7% reported not knowing the purpose of at least one of their medications, and only reading a portion of the PILs. Of the patients sampled, 56.7% said they read the side effects section of the PIL, 43.3% reported reading the uses, while 27% read the contraindications. The drug -interactions section was the least read, with only 18.9% reporting reading it. A total of 57% of the patients reported that they needed more information about their medications. Highly educated patients reported using the PIL, social media, family and friends, TV, and newspapers as sources of drug information at significantly higher rates than patients with lower levels of education. Healthcare professionals should assess patient comprehension and the need for additional drug information, especially among patients with low levels of education. Additionally, healthcare professionals should consider other information sources used by their patients.

## 1. Introduction

The health care in kingdom of Saudi Arabia (KSA) were one of many fields that ex-panded and improved under national transformative plan recently. The focus of pharma-ceutical services within the health service today is on providing effective pharmaceutical care. The community pharmacies were one of the frequently visited places by Saudi healthcare consumers for many reasons including dispensing their prescription, seek ad-vice related to their diseases and medications [1]. Because it’s convenient and easily ac-cessible by the consumers, the community pharmacies play crucial role in pharmaceutical care services offered to the patients in the country. A study conducted in Saudi Arabia reported that patient-centered care is a new concept in community pharmacies in the country and perceived that the knowledge and skills of community pharmacists are insufficient to provide effective patient-centered care [1]. However, the study acknowledged that chain community pharmacy groups in Saudi Arabia do provide diabetes education and other patient-centered care programs in some of their community pharmacies [2]. Few studies in the KSA have demonstrated that Saudi healthcare consumers appreciate community pharmacies providing patient-centered care, and they instead prefer to ask medication- and disease-related questions at community pharmacies over primary healthcare centers and hospitals because of their easy accessibility and convenient opening times [3,4].

Appropriate drug information is critical for proper drug use in pharmacy care practice. providing patients with education about their prescriptions and on proper medication administration has become an essential part of the pharmaceutical care process [5,6,7]. Inadequate patient education on drug therapy can result in therapeutic failure, disease recurrence, drug-induced side effects, and increased costs [7,8]. Patients’ emotional understanding and access to appropriate information should be prioritized when providing healthcare [9]. Drug information is especially important for equipping and preparing patients to take their medications correctly. According to studies, patients who receive accurate drug information better adhere to their drug treatment regimens [8]. Patient understanding of the purpose of their medications and fear of adverse effects can have a significant effect on medication adherence [10,11].

Previous research from various countries has shown that physicians and pharmacists are the most frequently used sources of drug information by patients [12,13,14]. The majority of patients appear to receive insufficient drug-related instructions from their doctors or pharmacists [15]. Furthermore, some patients may not understand all the information given by their healthcare professionals. As a result, these patients may look for alternative sources of Patient Information Leaflets (PILs) [12,13,16]. Written PILs are useful to a large proportion of the public [17]. Most PILs that come with medications contain several instructions: information on drug preparation, mechanism of action, pharmacokinetics, adult and pediatric dosages for various illnesses that can be treated, adverse effects, drug interactions, contraindications, cautions and warnings, a package of drug prescription, and ideal storage conditions [18]. Previous research found that patients also obtain drug information from other sources, including the media (TV, newspapers), their peers, and the Internet [12,13]. However, media coverage of medications may be insufficient in terms of the drugs’ benefits, risks, and costs [19]. This study’s objectives were to identify patients’ knowledge of prescribed medications, understand their desire for more information, and determine their sources of medication information in Saudi Arabia’s Qassim region.

## 2. Methods

### 2.1. Study Area and Period

The target population was adult and elderly patients with chronic illnesses receiving drugs from outpatient pharmacies in the Qassim Region, Saudi Arabia. The study was conducted between March 2018 and June 2018.

### 2.2. Study Design

A cross-sectional descriptive study was used to collect data from patients with chronic diseases visiting outpatient pharmacies in the Qassim region.

### 2.3. Study Population

Our target population was adult and elderly patients with chronic illnesses receiving drugs from outpatient pharmacies during the study. Patients who were 18 years of age or older were invited to take part in the study. Patients unable to complete the interview due to cognitive issues were excluded. The included age intervals were: below 25 years, 25–39 years, 40–50 years, 51–59 years, and 60 years or older.

### 2.4. Sample Size Determination and Sampling Method

Sample size was calculated using the following formula developed by Cochran [20]:

(1) $$N=\frac{Z^{2}\left(\mathrm{PQ}\right)}{D^{2}}=\frac{1.96^{2}\;\left(0.5*0.5\right)}{{\left(0.06\right)}^{2}}=\frac{0.9604}{0.0036}=266.6\;\approx \;270\;\mathrm{patients}$$

where N is the sample size, Z is the standard error associated with the selected level of confidence, P is the population proportion, Q = (1 − P), and D is the error of estimation (precision). The level of confidence used in this study was 95%. The Z value associated with 95% confidence was 1.96. The actual value of P was not known before the study; if P is unknown, researchers typically use 0.5 as an estimation, which made Q = 1 − 0.5 = 0.5. the error of estimation was 6%. 

The sample size was 270 patients. A convenience sampling technique was used, and only those who agreed to participate were included in the study.

### 2.5. Study Questionnaire

Data were collected using a questionnaire derived from the literature (permission was granted by the main author of Amin et al., 2011 [21]. The questionnaire was pretested on 18 patients (6% of the sample size) and then modified accordingly. The questionnaire investigated patients’ information, willingness for more information, and drug information sources beyond their healthcare providers. The first questions focused on patient’s knowledge of the purpose of their drug(s) and whether they needed more information about the drugs (Table 1). Subsequent questions were related to reading specific risk-related topics in package inserts, including side effects, contraindications, and drug interactions. Other questions included demographic data and drug information sources.

### 2.6. Data Collection

Data collection was carried out at outpatient pharmacies by fifth-year pharmacy students. A total of 270 patients with prescriptions for medications for chronic conditions were interviewed. The interviewers called on patients to take part in the study as they left the pharmacies after collecting their medications.

### 2.7. Data Analysis

SPSS version 20.0 software was used to analyze the data. Descriptive statistics (i.e., frequencies and percentages) were used to summarize participants’ responses to the questionnaire. Chi-square test was used to examine any associations that exist between categorical variables. A *p*-value of <0.05 was considered statistically significant.

### 2.8. Ethical Approval

The ethical approval for this study was obtained from the regional research ethics committee, Qassim Region (Reference no. 2018-02-10). The participants were informed with the aims of study and the data protection of the participants. Then, verbal consent was obtained from the patients to participate in the study. All participation was voluntary.

## 3. Results

A total of 270 outpatients from 19 pharmacies (16 community pharmacies and three outpatient pharmacies in three major hospitals in the area) were interviewed. The study population comprised 46.1% females and 53.9% males (Table 2).

The study population also included a range of education backgrounds, from elementary school to university. Table 2 shows that 43% of the study population had university education, 27% were high -school educated, and 30% had elementary education. Table 3 shows that more than half of our study sample (52.6%) reported regularly receiving 1–3 drugs, 27.8% received up to five drugs, 10.4% received up to seven drugs, and 9.2% were prescribed more than seven drugs. Perhaps unsurprisingly—given that our study focused on patients with chronic conditions—patients below 25 years of age constituted the smallest percentage of participants in our study (13%). Most of our study population consisted of patients aged 51–59 years (23%) and patients older than 60 years (22.2%). Patients between the ages of 25 and 39 years comprised 21.1% of our study population, while patients between the ages of 40 and 50 years comprised 20.7% of the study population, as shown in Table 2. Diagnoses and types of chronic diseases are also reported. diabetes and/or hypertension were the major illnesses in our sample population (40.3%). patients with GIT (gastrointestinal) disorders comprised only 10% of the population. other diagnosed diseases included heart diseases (4.4%), kidney diseases (3.3%), and allergies (5.6%). Patients with diseases not mentioned in the questionnaire had the option of choosing ‘other’. they were subsequently asked to state the type(s) of chronic diseases they were suffering from. These patients comprised 36.4% of the study population (Table 3).

The interviewers assessed the patients’ perceived understanding of the purpose of the drugs they were taking. A high number of patients (70%) reported knowing the purpose of the drugs; however, 14.1% of the study population did not know the purpose of the drugs they were consuming, and 15.6% had an idea of the purpose of some, but not all, of the drugs, as indicated in Table 4. The results in Table 4 also show how sex, education, diagnosis, number of drugs, and age affect knowledge of the purpose of medications and the need for additional information. The table shows that both male and female patients had a relatively high level of knowledge of the purpose of their drugs—67.6% and 72.8%, respectively. It was clear that more highly educated patients had a higher likelihood of knowing the purpose of the drugs they were taking (*p <* 0.05). For instance, 87% of patients who reported having university education knew the purpose of their prescribed drugs; 64.4% of high -school -educated patients reported knowledge of their drugs, while primary -school -educated patients were the least likely to know the purpose of their drugs (49.4%). Regarding diagnosis, the results show that 67.4% of patients with diabetes identified the purpose of their prescribed medications, 68.2% of hypertensive patients identified the purpose of their respective medications, and 83.3% of patients with heart diseases identified the purpose of their medications. Other notably high proportions of patients with self-reported drug knowledge included patients with GIT disorders and allergies—85.2% and 86.7%, respectively. With respect to the number of drugs, 76.1% (108) of the patients taking 1–3 medications identified the purpose of all their medications, but when patients were taking more than seven medications, this proportion reduced (44%). Table 4 clearly shows that the younger the patient, the higher the likelihood of them knowing the purpose of their drugs: 94.2% of patients under the age of 25 years reported that they knew the purpose of the drugs they were taking; however, this decreased as the patients grew older. The 25–39 and the 40–50 age groups reported knowing the purpose of their medications 82.6% and 73.2% of the time, respectively. In the 51–59 age group, this number was 59.7%. Finally, the oldest age group (the 60 years or older age group) reported this 51.7% of the time.

Another aspect of the study was to gauge patients’ need for more information about their medications. Table 4 shows that there was little difference between the sexes, as most of both male and female patients reported a need for more drug information—71.7% and 75.2%, respectively. Among university educated patients, 82.6% wanted more drug information, whereas 74% of high-school-educated patients and 55.5% of primary-school-educated patients expressed this desire (*p <* 0.05). diabetic and hypertensive patients had similar views regarding the desire for more information, at 69.8% and 68.2%, respectively. Patients who had both diabetes and hypertension concurrently reported a need for more information about their prescribed medications 70.4% of the time. Notably, patients suffering from GIT disorders reported the highest need for more information (88.9%), followed by patients with heart disease (83.3%). Of the Other diagnoses, 66.7% of patients with kidney disease and 66.7% of patients with allergies reported a need for more information.

The results in Table 5 show that almost half of the patients (49.6%) reported that they read the Package Information Leaflets (PILs) sometimes, while 25.2% reported always reading them, and 25.2% reported never reading the PILs.

The results in Table 5 indicate that 25.56% of the patients did not read any of the adverse effects, uses, drug interactions, or contraindication sections of the package inserts. As Table 6 clearly shows, 56.5% of the patients said they read the side effects portion of the PIL, 43.7% reported reading the topic on uses, 27% read about the contraindications, while drug interactions were read the least number of times, by only 18.8% of the patients.

The results in Table 7 show that the most frequently used source of drug information was the Internet (58.75%), followed by family and friends (47%) (*p <* 0.05). TV as a source of information was used by only 14.2% of the patients, while newspapers were the least used source of information, with only 4.4% of patients relying on them for drug information. Table 7 shows, as expected, that patients with university education mostly used the internet, with 79.3% reporting this as a source of drug information. More than half of patients with a high school education used the Internet (57.5%), while 29.6% of patients with elementary -level education reported using the internet as a source of drug information. There was a significant proportional relationship between using the Internet (which generally requires at least an adequate knowledge of English) and education level: patients with a higher level of education were more likely to use the internet to obtain additional drug information. On the relationship between age and use of the Internet, a high percentage of patients aged 25–39 years reported using the internet (72%). This was also the case in the 40–50 age group (71.4%). More than half (54.8%) of patients aged 51–59 used the Internet. Among patients below 25 years of age, 68.6% reported using the Internet. However, the percentage of older patients (aged 60 years or over) using the Internet was lower than the other age groups, with just 31.7% reporting using the Internet to obtain drug information.

## 4. Discussion

Prescriptions are the most common therapeutic interventions in medical practice. As a result, reliable drug information is essential for pharmaceutical care. Barber et al. [22] found that a relatively high percentage of drug information needs in their study had not been met. In that study, 52% of elderly patients aged 75 years or older who were on new medications for chronic conditions desired more information on their medications after four weeks. This was slightly lower than the percentage of patients who were 60 years or older (63.3%) who said they needed more drug information in our study.

Furthermore, Davis et al. [23] indicated that patients with chronic diseases who had a lower level of education were more likely to misunderstand the information written on medication labels. The reason patients with primary school education reported poor understanding and a lower need for more drug information, as shown in Table 4, was that they were less likely to read the Patient Information Leaflet (PIL) topics compared with university educated patients. This finding is in agreement with the findings of other studies [17,24]. Another possible cause is that patients with a low level of education who read the PILs may have difficulty understanding them. 

With respect to other sources of drug information, patients with a lower level of education were less likely to use TV, newspapers, and the internet as sources of medication information compared with patients with secondary school and university education. These findings are consistent with those of Brodie et al. [24].

Based on our findings, patients with a lower level of education depend mainly on periodic pharmacy visits to evaluate and discuss issues relating to their understanding of the purpose, side effects, and drug interactions of their medications.

Previous research indicates that a substantial number of patients think that their healthcare providers fail to adequately inform them about drug treatments [15]. Furthermore, many serious medication-related questions arise the moment the patient has left the clinic or pharmacy. Approximately 47% of our study population relied primarily on unreliable sources of information, such as friends and family. This finding was relatively consistent with the 40% found in an Iranian study [25]. Previous research has also found that, in addition to family and friends, TV is a significant source of drug information [25]. This lack of consistent access to information could have serious health consequences, and therefore needs to be addressed and resolved by health policymakers and other relevant stakeholders.

In some nations, many patients search for medication information on the Internet [24]. In our study, 58.5% of patients obtained drug information from the Internet. However, in another study [25], the Internet played an insignificant role. In a study undertaken in Egypt by Amin et al. (2011) [21], the Internet was the least-used source of information, with only 12% reporting its use, whereas reliance on family and friends, at 48.6%, was consistent with our finding.

The gap in Internet usage in the two studies is interesting and somewhat logical because it was estimated that 35.6% of the Egyptian population had access to and used the Internet at the time of the study by Amin et al. at 2011 [21], whereas, at the time of our study at 2018, a much larger proportion of the Saudi population had access to the Internet.

The instructions attached to the drug container and PILs are the most reliable sources of drug information. Several studies have found that written information effectively improves patient compliance [26]. Our study found that 25.2% of patients reported always reading the package insert, which was in agreement with another study where 27% of the patients read the medication information leaflets [24]. However, in the study conducted in Iran [25], only 14% of the patients frequently used PILs as sources of medication information. Primary-school-educated patients were less likely to read PILs.

A lack of communication between clinicians or pharmacists and their patients, as well as only occasionally reading PILs, may make patients turn to untrustworthy sources of information such as family and friends. These patients are more susceptible to medication errors that result in severe health issues [9,27]. Therefore, carefully designed, impartial, scientifically reliable, and updated PILs written in a legible format that is understandable to the average patient are of utmost importance [27].

The number of drugs taken by a patient is an important parameter for physicians and pharmacists to consider when patients require more drug information. According to the study’s findings, the more medications patients took, the less likely they were to understand the reasons for taking them and the less likely they were to read about the adverse effects, uses, drug interactions, and contraindications contained in the PILs. Patients who had more medications were also more likely to need more drug information when they left the pharmacy. Davis et al. (2006) reported that a greater number of prescription medications used by patients was significantly associated with misunderstanding the instructions on medications label [23]. It is important for patients taking five or more medications to be aware that there is a higher risk of drug interactions.

Our results indicate that patients were more likely to read the side effects than the uses, drug interactions, or contraindications. Side effects have been reported to be the most frequently read topic in written drug information [27,28]. Vander Stichele et al. [27] found that patients focused more on side effects and less on contraindications, which is consistent with our results. According to Raynor et al. [28], 12% of those who received a PIL did not read it because they did not see it. Van Haecht et al. [29] reported no sex differences in PI reading; however, several other studies have found that women read more drug information leaflets than men [27]. 

There was a small difference in the frequency of PIL reading between the two sexes in our study. It is essential to take the study’s country into account when interpreting these findings. The patient’s age also influenced the perception of the necessity for medication information and the use of other drug information sources.

Duggan and Bates [30] reported an inverse relationship between age and the desire for information on medication. This contradicted our finding of unmet drug information needs, which were highest among those under the age of 25 years, and then gradually declined in patients aged 25–39, 40–50, and 51–59 years. Furthermore, younger people used the Internet to obtain drug information more frequently. Elderly patients were found to be less familiar with modern drug information sources such as the Internet [6]. While receiving the same quantity of information, one patient might feel that it is inadequate and another patient might feel that it is adequate. This distinction will have an effect on both satisfaction with current information and the active search for additional information. The need and desire for extra information may be influenced by the patient’s state and disease, such as the type of diabetes or the severity of hypertension.

The main limitation of this study was the use of convenience sampling to recruit pa-tients. “ Do you think you need to know more about the drugs you’re taking?” is a question that is positively framed. This question may be prompted patients to answer positively. Also, our study only included patients from the Qassim area and conducted in a specific time so, the findings obtained from the population reflect specific time. More re-search should be conducted to study medication practices and behaviors among patients with multiple chronic diseases in different areas of Saudi Arabia to compare the various trends. 

## 5. Conclusions

After leaving the pharmacy, many patients with chronic diseases stated that they did not grasp the intended use of at least one of their drugs, and many of them said they wanted more information about the drugs. Based on this information, it is clear that many patients in our study did not understand or may not have received adequate information from clinicians or pharmacists; thus, their understanding and desire for information were not adequately assessed. Since pharmacists are the last point of contact for patients, they should assess patient understanding and the need for additional drug information. Patients with a university or secondary school education often rely on other sources of information, such as PILs, to meet their medication information needs, compared with patients with primary school education. These findings imply that healthcare providers should focus on patient education and understanding, particularly among patients with primary school education and those taking multiple medications. Healthcare decision-makers should consider education level when developing accurately designed, impartial, scientifically reliable, and updated PILs written in a legible format that is understandable to the average patient. Healthcare professionals who deliver services should suggest recognized resources suitable for a patient’s disease and level of education, while also encouraging patients to discuss any related issues and concerns raised by other resources.

## Figures and Tables

**Table 1:** Survey questions.

Question	Answer Choices
What are your diagnoses?	
Do you know the purpose of the drugs you are consuming?	(1) Yes (2) No (3) I
	know the purpose
	of some of them
Do you think you need to know more about the medications you are consuming?	(1) Yes (2) No
What do you read on the package insert?	
a. Side effects	(1) Yes (2) No
b. Uses	(1) Yes (2) No
c. Drug interactions	(1) Yes (2) No
d. Contraindications	(1) Yes (2) No
Other than the pharmacist and the doctor, what are your sources of information about drugs?	
a. Newspapers	(1) Yes (2) No
b. Internet	(1) Yes (2) No
c. TV	(1) Yes (2) No
d. Family and friends	(1) Yes (2) No

**Table 2:** Patients’ demographic data.

Characteristics	N = 270 (%)
Age	
Less than 25 years	35 (13)
25–39	57 (21.1)
40–50	56 (20.7)
51–59	62 (23)
Equal or more than 60	60 (22.2)
Gender	
Male	145 (53.7)
Female	125 (46.3)
Education level	
University	116 (43)
High Secondary	73 (27)
Primary school	81 (30)

**Table 3:** Diagnoses and the number of drugs used by patients in the study.

Characteristics	N = 270 (%)
Diagnoses	
Diabetes	43 (15.9)
Hypertension	22 (8.1)
Diabetes and hypertension	44 (16.3)
Heart diseases	12 (4.4)
Kidney diseases	9 (3.3)
Allergy	15 (5.6)
GIT disorder	27 (10)
Other	98 (36.4)
No. of drugs	
1 to 3 drugs	142 (52.6)
3 to 5 drugs	75 (27.8)
5 to 7 drugs	28 (10.4)
More than 7 drugs	25 (9.2)

**Table 4:** Knowledge of the purpose of medications and the need for additional information (N = 270).

Characteristics	Responses for Knowing Purposes of Drugs *N* (%)			Patients Needing More Information about Medication (s) *N* (%)
	Yes	No	Some	
Sex				
Male	98 (67.6)	21 (14.5)	26 (17.9)	104 (71.7)
Female	91 (72.8)	17 (13.6)	17 (13.6)	94 (75.2)
Education				
University	101 * (87)	5 (4.3)	10 (8.6)	100 * (86.2)
High school	47 * (64.4)	17 (23.3)	9 (12.3)	54 * (74)
Primary	40 * (49.4)	17 (21)	24 (29.6)	45 * (55.5)
Diagnosis				
Diabetes	29 (67.4)	7 (16.3)	7 (16.3)	30 (69.8)
Hypertension	15 (68.2)	5 (22.7)	2 (9.1)	15 (68.2)
Diabetes and Hypertension	28 (63.6)	9 (20.5)	7 (15.9)	31 (70.4)
Heart Diseases	10 (83.3)	0(0.0)	2 (16.7)	10(83.3)
Kidney Diseases	6 (66.7)	2 (22.2)	1 (11.1)	6 (66.7)
Allergy	13 (86.7)	0 (0.0)	2 (13.3)	10 (66.7)
GIT Disorders	23 (85.2)	1 (3.7)	2 (7.4)	24 (88.9)
Others				
No. of drugs				
1–3	108 (76.1)	15 (10.6)	18 (12.7)	102 (71.8)
3–5	53 (70.7)	12 (16)	10 (13.3)	62 (82.7)
5–7	17 (60.7)	6 (21.4)	5 (17.9)	20 (71.4)
More than 7	11 (44)	5 (20)	9 (36)	15 (60)
Age				
Less than 25 years	33 (94.2)	2 (5.8)	0 (0)	31 * (88.6)
25–39	47 (82.6)	4 (7)	5 (8.7)	50 * (87.7)
40–50	41 (73.2)	5 (8.9)	10 (17.8)	45 * (80.6)
51–59	37 (59.7)	10 (16.1)	15 (24.2)	35 * (56.4)
Equal or more than 60	31 (51.7)	17 (28.3)	12 (20)	38 * (63.3)
Total	189 (70)	38 (14.1)	42 (15.6)	154 (57)

* Significance set at *p* < 0.05. results obtained using Chi-square test.

**Table 5:** Patients reporting reading package inserts (N = 270).

Characteristics	Responses for Reading the Package Insert *N* (%)		
	Yes	No	Sometimes
Gender:			
Male	37 (25.5)	43 (29.6)	65 (44.8)
Female	31 (24.8)	26 (20.8)	68 (54.4
Education:			
University	35 (30.2)	12 (10.3)	69 (59.5)
High school	20 (27.4)	29 (39.7)	34 (46.6)
Primary	13 (16)	38 (47)	30 (37)
Diagnosis:			
Diabetes	13 (30.2)	15 (34.9)	15 (34.9)
Hypertension	7 (31.8)	4 (18.2)	11 (50)
Diabetes and Hypertension	7 (15.9)	13 (29.5)	24 (54.5)
Heart Diseases	6 (50)	1 (8.3)	5 (41.7)
Kidney Diseases *	4 (44.4)	3 (33.3)	2 (22.2)
Allergy	2 (13.3)	5 (33.3)	8 (53.3)
GIT Disorders	9 (33.3)	2 (7.4)	16 (59.3)
Others	20 (20.4)	26 (29.2)	52 (58.4)
Age:			
Less than 25 years	12 (34.3)	2 (5.7)	21 (60)
25–39	17 (29.8)	9 (15.8)	31 (54.4)
40–50	18 (32.1)	12 (21.4)	26 (46.4)
51–59	11 (17.7)	20 (32.3)	31 (50)
Equal or more than 60	10 (16.7)	26 (43.3)	24 (40)
Total	68 (25.2)	69 (25.6)	133 (49.2)

* Significance set at *p* < 0.05. results obtained using Chi-square test.

**Table 6:** Responses on patients reading package inserts.

Characteristics	Reported Response for Reading Package Insert			
	Side Effect	Uses	Drug Interactions	Contraindications
Gender				
Male	89 (61.4)	60 (41.4)	24 (16.6)	33 (22.8)
Female	64 (51.6)	57 (46)	26 (21)	39 (31.5)
Education level				
University	84 * (73)	60 * (52.2)	28 * (24.3)	39 * (33.9)
High school	39 * (54.2)	33 * (45.8)	14 * (19.4)	20 * (27.8)
Primary	30 * (37.5)	24 * (30)	9 * (11.25)	14 * (17.5)
Diagnosis				
Diabetes	20 * (46.5)	9 * (20.9)	4 (9.3)	9 (20.9)
Hypertension	9 * (40.9)	9 * (40.9)	1 (4.5)	6 (27.3)
Diabetes and hypertension	25 * (56.8)	21 * (47.7)	11 (25)	10 (22.7)
Heart diseases	10 * (83.3)	5 * (41.7)	3 (25)	4 (33.3)
Kidney diseases	5 * (55.6)	4 * (44.4)	1 (11.1	5 (55.6)
Allergy	9 * (60)	5 * (33.3)	2 (13.3)	3 (20)
GIT disorder	22 * (81.5)	12 * (44.4)	5 (18.5)	7 (25.9)
Others	53 * (54.1)	55 * (88.7)	24 (24.5)	29 (29.6)
No. of drugs				
1 to 3	79 (55.6)	57 (40.1)	18 * (12.7)	39 (27.5)
3 to 5	44 (58.7)	35 (46.7)	15 * (20	21 (28)
5 to 7	15 (53.6)	11 (39.3)	8 * (28.6)	9 (32.1)
More than 7	15 (60)	14 (56)	10 * (40)	4 (16)
Age (years)				
Less than 25 years	25 * (71.4)	18 (51.4)	8 (22.9)	9 (25.7)
25 to 39	36 * (63.2)	24 (42.1)	12 (21.1)	20 (35.1)
40 to 50	36 * (64.3)	31 (55.4)	6 (10.7)	14 (25)
51 to 59	29 * (46.8)	19 (30.6)	13 (21)	13 (21)
Equal or more than 60	27 * (45)	25 (41.7)	12 (20)	17 (28.3)
Total	153 (56.7)	117 (43.3)	51 (18.9)	73 (27)

* Significance set at *p* < 0.05. results obtained using Chi-square test.

**Table 7:** Patients’ other sources of drug information.

Characteristics	Other Sources of Information about Drugs *N (%)*			
	Newspapers	Internet	TV	Family and Friends
Sex				
Male	8 (5.5)	81 (55.9)	25 (17.2)	68 (46.9)
Female	4 (3.2)	77 (61.6)	14 (11.2)	59 (47.2)
Education				
University	3 (2.6)	92 * (79.3)	13 (11.2)	42 (36.2)
High school	4 (5.5)	42 (57.5)	12 (16.4)	39 (53.4)
Primary	5 (6.2)	24 (29.6)	14 (17.3)	46 (56.8)
Diagnosis				
Diabetes	3 (7)	26 * (60.5)	10 (23.3)	16 (37.2)
Hypertension	2 (9.1)	15 * (68.2)	3 (13.6)	7 (31.8)
Diabetes and Hypertension	4 (9.1)	21 * (47.7)	7 (15.9)	25 (56.8)
	0 (0.0)	4 * (33.3)	1 (12)	7 (58.3)
Heart Diseases	0 (0.0)	4 * (44.4)	2 (22.2)	5 (55.5)
Kidney Diseases *	1 (6.7)	7 * (46.7)	2 (13.3)	5 (33.3)
Allergy	0 (0.0)	56 * (57.1)	1 (3.7)	7 (25.9)
GIT Disorders	3 (3.1)		13 (13.3)	55 (56.1)
Others				
No. of drugs				
1 to 3	6 (4.2)	92 * (64.8)	24 (19.9)	53 * (37.3)
3 to 5	5 (6.7)	46 * (61.3)	9 (12)	38 * (50.7)
5 to 7	0	12 * (42.9)	2 (7.1)	22 * (78.6)
More than 7	2 (8)	8 * (32)	4 (16)	14 * (56)
Age				
Less than 25 years	2 (5.7)	24 * (68.6)	2 (5.7)	19 * (54.3)
25–39	4 (7)	41 * (72)	7 (12.3)	16 * (28.1)
40–50	1 (1.8)	40 * (71.4)	8 (14.3)	25 * (44.6)
51–59	1 (1.6)	34 * (54.8)	9 (14.5	29 * (46.8)
Equal or more than 60	4 (6.7)	19 * (31.7)	13 (21.7)	38 * (63.3)
Total	12 (4.4)	158 (58.5)	39 (14.4)	127 (47)

* Significance set at *p* < 0.05. Results obtained from Chi-square test.

## Data Availability

The datasets used for this study were available from the corresponding author on reasonable request.
